# Flint River Medical Association

**Published:** 1876-08

**Authors:** 


FLINT RIVER MEDICAL ASSOCIATION.—We are pleased to see in different portions of our State the organization of local medical associations. There is nothing, in our opinion, which does so much to elevate the tone of the profession, by insuring pleasant relations, both social and professional, as such organizations. They will ever have our hearty support, and at any and all times it will give us pleasure to serve them. The following report of the organization was received too late for the July number of our JOURNAL. The proceedings of this association will appear from time to time in the Journal.

OFFICE FLINT RIVER MEDICAL ASSOCIATION,
MONTEZUMA, GA., May 10, 1876.

Pursuant to a call made to the physicians of Macon, Dooly, and adjoining counties to meet in the Town Hall at Montezuma, Ga., on May the 10th, at 2 o'clock P.M., quite a respectable number met and organized a medical society.

On motion of Dr. J. W. Vinson, Dr. J. M. R. Westbrook was called to the chair.

On motion of Dr. A. A. Watts, Dr. R. O. Engram was requested to act as Secretary.

After the object of the meeting was explained by Dr. J. M. R. Westbrook, the following resolutions were adopted:

WHEREAS, We have met as a body of physicians, to organize a medical society for our mutual improvement and benefit, and for cultivating more intimate associations and social relations between the members of our profession; and whereas, the profession is sadly in need of organization looking to these ends; therefore,

*Resolved*, That we do organize a medical society, to be known as the "Flint River Medical Association."

*Resolved*, In order that we may become fully organized, that for the present the officers of this Association shall consist of a President, two Vice-Presidents, Treasurer, and Secretary.

On motion, Dr. J. M. R. Westbrook was elected permanent President; Dr. A. A. Watts 1st, and Dr. J. L. Gibson 2d, Vice-Presidents; Dr. J. W. Vinson Treasurer, and Dr. R. O. Engram Secretary.

On motion, a committee of three were appointed to draft a constitution and by-laws to govern this Association, consisting of Drs. Mason, Gibson, and Engram.

On motion, it was agreed that this Association meet, for the present, in the town of Montezuma, on the second and fourth Wednesdays in each month, at 2 o'clock P.M.

On motion, the Secretary was requested to invite all regular physicians, in good standing with our profession, to meet with the Association on Wednesday, May 24th, 1876.

*Resolved*, That it shall be the duty of each member of this Association to report every case that comes within his knowledge which has been maltreated by irregular and unprofessional persons; and it shall be the duty of this Association, through its President, to appoint a committee of six, in addition with himself as chairman of such committee, to obtain permission of persons who are duped and maltreated by quacks, charlatans, and persons claiming the honors of our profession, to investigate such malpractice, and report the same to this Association for their consideration, that they may determine whether they shall protect their suffering fellow-beings from the empiricisms of charlatans and the impositions of quacks.

R. O. ENGRAM.

